# Efficacy of psychosocial interventions for Autism spectrum disorder: an umbrella review

**DOI:** 10.1038/s41380-022-01670-z

**Published:** 2022-07-05

**Authors:** Corentin J. Gosling, Ariane Cartigny, Baptiste C. Mellier, Aleix Solanes, Joaquim Radua, Richard Delorme

**Affiliations:** 1 Paris Nanterre University, DysCo Laboratory, F-92000 Nanterre, France; 2 Université de Paris, Laboratoire de Psychopathologie et Processus de Santé, F-92100 Boulogne-Billancourt, France; 3 Centre for Innovation in Mental Health (CIMH), School of Psychology, Faculty of Environmental and Life Sciences, University of Southampton, Southampton, UK; 4 Department of Child and Adolescent Psychiatry, Robert Debré Hospital, APHP, Paris, France; 5 Imaging of Mood- and Anxiety-Related Disorders (IMARD) Group, Institut d’Investigacions Biomèdiques August Pi i Sunyer (IDIBAPS), CIBERSAM, Barcelona, Spain; 6 Department of Psychosis Studies, Institute of Psychiatry, Psychology, and Neuroscience, King’s College London, London, UK; 7 Department of Clinical Neuroscience, Centre for Psychiatric Research and Education, Karolinska Institutet, Stockholm, Sweden; 8 Human Genetics and Cognitive Functions, Institut Pasteur, Paris, France

**Keywords:** Autism spectrum disorders, Psychology

## Abstract

**Introduction:**

The wide range of psychosocial interventions designed to assist people with Autism Spectrum Disorder (ASD) makes it challenging to compile and hierarchize the scientific evidence that supports the efficacy of these interventions. Thus, we performed an umbrella review of published meta-analyses of controlled clinical trials that investigated the efficacy of psychosocial interventions on both core and related ASD symptoms.

**Methods:**

Each meta-analysis that was identified was re-estimated using a random-effects model with a restricted maximum likelihood estimator. The methodological quality of included meta-analyses was critically appraised and the credibility of the evidence was assessed algorithmically according to criteria adapted for the purpose of this study.

**Results:**

We identified a total of 128 meta-analyses derived from 44 reports. More than half of the non-overlapping meta-analyses were nominally statistically significant and/or displayed a moderate-to-large pooled effect size that favored the psychosocial interventions. The assessment of the credibility of evidence pointed out that the efficacy of early intensive behavioral interventions, developmental interventions, naturalistic developmental behavioral interventions, and parent-mediated interventions was supported by suggestive evidence on at least one outcome in preschool children. Possible outcomes included social communication deficits, global cognitive abilities, and adaptive behaviors. Results also revealed highly suggestive indications that parent-mediated interventions improved disruptive behaviors in early school-aged children. The efficacy of social skills groups was supported by suggestive evidence for improving social communication deficits and overall ASD symptoms in school-aged children and adolescents. Only four meta-analyses had a statistically significant pooled effect size in a sensitivity analysis restricted to randomized controlled trials at low risk of detection bias.

**Discussion:**

This umbrella review confirmed that several psychosocial interventions show promise for improving symptoms related to ASD at different stages of life. However, additional well-designed randomized controlled trials are still required to produce a clearer picture of the efficacy of these interventions. To facilitate the dissemination of scientific knowledge about psychosocial interventions for individuals with ASD, we built an open-access and interactive website that shares the information collected and the results generated during this umbrella review.

**Pre-registration:**

PROSPERO ID CRD42020212630.

## Introduction

Autism Spectrum Disorder (ASD) is a neurodevelopmental disorder that is characterized by social communication deficits which are associated with restricted and repetitive patterns of behaviors and interests that interfere with quality of life [1]. Numerous interventions have been designed to assist people with ASD and their families [2–4]. However, the wide range of psychosocial interventions makes it difficult to compile and hierarchize the scientific evidence supporting the efficacy of these interventions, resulting in insufficient dissemination of the scientific evidence which supports their efficacy [5]. Individuals with ASD are sometimes engaged in treatments for which there is weak empirical evidence or, more dramatically, that can result in harmful consequences [6, 7]. The present umbrella review aims to provide a clearer picture of the efficacy of the psychosocial interventions on both core and related ASD symptoms.

A large variety of psychosocial interventions have been developed to improve ASD symptoms across the lifespan, and numerous clinical trials have been conducted to explore their efficacy [8, 9]. The first interventional approach to be investigated in clinical studies were behavioral interventions, which are based on operant learning theories [10]. Promising results from the initial clinical studies that delivered these behavioral techniques at a very high intensity (referred to as early intensive behavioral interventions; EIBI), led to their widespread adoption in clinical practice [11–13]. In contrast to behavioral interventions, developmental interventions (DEV) are based on constructivist models [14]. DEV focus on supporting children’s social interactions with others during daily life activities, e.g., play [15]. Recently, the new category of naturalistic behavioral developmental interventions (NDBI) has emerged to describe practices that are rooted in both behavioral and developmental theories [16]. NDBI employ a diversity of behavioral techniques that promote the emergence of developmentally appropriate skills in a natural setting [17]. Social skills groups (SSG) are another psychosocial intervention that have received substantial experimental support [18]. Delivered in a group setting, SSG seek to improve social skills by combining structured learning of prosocial behaviors with drill and practice exercises during and between sessions. Besides, cognitive-behavioral therapy (CBT), which has shown robust efficacy on symptoms of various mental health conditions [19], has also been applied to individuals with ASD [20]. Based on a combination of principles from both behavioral and cognitive sciences, CBT target coping skills to enable individuals to modify their maladaptive thoughts, emotions, and behaviors. Unlike the approaches mentioned above, the Treatment and Education of Autistic and Related Communication-Handicapped Children (TEACCH) focuses on structuring the environment of individuals with ASD [21, 22]. This program was developed to create a highly structured learning environment which capitalizes on the relative strengths of individuals with ASD, e.g., their visual skills. Finally, rather than the intervention content, the mode of delivery has also been the subject of clinical assessment for individuals with ASD. In particular, many studies explored the efficacy of parent-mediated interventions (PMI) [23] and technology-mediated interventions (TECH) [24].

When it comes to assessing the efficacy of a drug or a non-drug intervention, systematic reviews and meta-analyses are robust tools to improve clinical decision-making [25]. However, as with any experimental study, these studies are also prone to inconsistency and methodological biases, which may produce divergent conclusions by several reviews or meta-analyses on the same topic [26]. For example, two meta-analyses in the field of ASD published in 2009, which explored the efficacy of EIBI on intelligence quotient, showed either a large and statistically significant pooled effect size in favor of EIBI [27] or a small and non-significant pooled effect size [28]. These discrepancies strongly reinforce the uncertainty for health providers and patients in selecting the optimal therapeutic strategy. Umbrella reviews are additional tools of evidence synthesis that have emerged in recent years to overcome the methodological limitations of meta-analyses [29, 30]. Umbrella reviews compile evidence from a large body of information by assessing the credibility of multiple meta-analytic results in a consistent, transparent, and reproducible framework [31]. In particular, umbrella reviews aim to direct clinical decision-makers to current best evidence relevant to a specific decision [32].

The objective of the present study was to perform an umbrella review that provided additional evidence on the efficacy of psychosocial interventions on both core and related ASD symptoms. We performed an algorithmic assessment of the credibility of the evidence that supported the efficacy of different psychosocial interventions using objective, transparent and reproducible criteria. We also critically assessed the methodology used in the meta-analyses to direct readers to best evidence. In parallel, we built an open-access online interactive resource that contains all of the information that we collected and all results that we generated for this umbrella review (Evidence-Based Interventions for Autism: Clinical Trials [EBIA-CT] database: https://www.ebiact-database.com). In summary, the present work aimed to provide a more reliable and accessible source of evidence-based information about the efficacy of psychosocial interventions in individuals with ASD [33, 34].

## Methods

### Search strategy and eligibility criteria

Reporting of this umbrella review followed the Preferred Reporting Items for Systematic Reviews and Meta-Analyses (PRISMA) guidelines [35] and its completion followed the most recent guidelines for umbrella reviews [36, 37]. This umbrella review was pre-registered on PROSPERO (ID CRD42020212630). The complete PRISMA checklist and the deviations from protocol are available online (Supplementary Tables S1 and S2, respectively).

Two authors (CJG and AC) searched five databases (Medline, EMBASE, CENTRAL, CINAHL, and PsycINFO) until October 1, 2021 using search terms related to two constructs: ASD and systematic review. Adaptations of previously validated search filters developed by the Hedges teams to retrieve systematic reviews and meta-analysis were used (see full strategies in Supplementary Text S3) [38–41]. No restrictions were made based on language or date of publication. Titles and abstracts were screened independently and, for articles that were deemed to be eligible, full texts were downloaded and assessed independently for inclusion in final analyses. A senior author (RD) resolved conflicts between CJG and AC. References in included articles were also searched.

We included systematic reviews coupled with a meta-analysis of at least two controlled clinical trials (CCTs) that assessed the efficacy of psychosocial interventions on ASD symptoms in participants with ASD. A review was considered as systematic if labeled as such or if searches of scientific databases were performed in combination with explicit inclusion/exclusion criteria. The definition of ASD followed those used by primary authors and was in line with Diagnostic and Statistical Manual of Mental Disorders (DSM)-III, DSM-IV, DSM-IV-TR, DSM-5, International Classification of Diseases (ICD)−9, or ICD-10. There were no exclusion criteria regarding ages of participants. Meta-analyses which focused on the same type of intervention and on the same outcome but in distinct age groups (pre-school children: a mean age ranging from 0 to 5 years; school-aged children: a mean age ranging from 6 to 12 years; adolescents: a mean age ranging from 13 to 19 years; adults: a mean age > = 20 years) were reported separately. We included meta-analyses of both randomized and non-randomized controlled trials (RCTs and NRCTs, respectively) since we anticipated that many interventions were not assessed by RCTs. We were concerned that synthesizing evidence only from RCTs may lead to an incomplete picture of the efficacy of psychosocial interventions in people with ASD. However, a sensitivity analysis restricted to RCTs was conducted (see Supplementary Results S2.A).

Based on several seminal textbooks [3, 4, 42, 43], we classified an intervention as psychosocial if assessing the efficacy of: (i) EIBI, (ii) NDBI, (iii) DEV, (iv) SSG, (v) PMI, (vi) CBT, (vii) TECH, and (viii) TEACCH. A description of each of these interventions can be retrieved from https://ebiact-database.com/interventions.html. Meta-analyses that pooled together trials which assessed the efficacy of at least two intervention types were excluded. An exception was made for PMI and TECH, since the focus of these intervention types was more on the delivery modalities than on content, and because readers may be interested specifically in the method used to deliver the intervention irrespective of the specific type of approach used. However, readers should be aware that there is a substantial overlap between PMI and NDBI/DEV, as parents are typically highly involved in the delivery of the intervention in NDBI and DEV. Meta-analyses of pharmacological interventions, occupational therapies, complementary and alternative medicine, or lifestyle interventions were not considered in this study.

Three of the eight pre-specified outcomes directly concerned core ASD symptoms: (i) overall ASD symptom severity, (ii) social communication deficit, and (iii) restrictive/repetitive behaviors or interests. We also considered five additional outcomes related to the main characteristics strikingly associated with ASD symptoms: (iv) cognitive global abilities (intelligence quotient, IQ), (v) adaptative behaviors, (vi) expressive language skills, (vii) receptive language skills, and (viii) the disruptive behaviors associated with ASD. We comprehensively extracted the data of all meta-analyses when specific articles reported several independent meta-analyses assessing the efficacy of the same intervention on the same clinical outcome. We resumed these meta-analyses into a unique pooled effect size using standard aggregating procedures (that are described further in the data analysis section below). The results of each individual meta-analysis are presented in Supplementary Results S2.C.

To handle overlapping meta-analyses, i.e., two independent reports that assessed the efficacy of similar interventions on equivalent samples and outcomes, we first selected all the overlapping meta-analyses that were published after January 1st, 2016 and then selected the meta-analysis with the highest methodological quality (see data extraction below). The concordance between all overlapping meta-analyses was assessed in a sensitivity analysis (Supplementary Results S2.B). Moreover, we located several meta-analyses of a brand-name or specific intervention, e.g., a meta-analysis of Early Start Denver Model, while more comprehensive meta-analyses on the same intervention type were also available, e.g., a meta-analysis pooling together several NDBI. In these cases, we favored the more comprehensive meta-analysis, but we reported the results of the brand-name or specific intervention in Supplementary Results S2.B.

### Data extraction

For each trial reported in a meta-analysis, two author pairs (CJG and AC or CJG and BCM) independently extracted information regarding participants (the number of participants, their mean age or age range, their mean total IQ [verbal and non-verbal IQ or developmental quotient were used as proxies when total IQ was not available], total IQ range, and the sex ratio); interventions (study design, subtype of intervention, use of assistance/mediation during the intervention, setting of the intervention [e.g., clinic], type of practitioner delivering the intervention [e.g., educator], mean hours per week of intervention, and mean duration of the trials in months); outcomes (category of the outcome [e.g., improvement of the total IQ], the method used to assess the outcome [e.g., questionnaire], and the tool name); design of studies (NRCT vs. RCT); type of control group (treatment as usual, eclectic, waiting list/delayed, or active control treatment); risk of bias; and effect size (effect size metrics, value, and 95% confidence interval, standard error, or variance).

The AMSTAR-2 tool was used to assess the methodological quality of each meta-analysis retained in primary analyses [44]. Scoring was made independently by two authors pairs (CJG and AC or CJG and BCM). Five core criteria of AMSTAR-2 were used to select the meta-analysis with the highest methodological quality in the presence of overlap: the presence of a priori research design, the quality of search characteristics, the independence in study selection and data extraction, and the assessment of the risk of bias in the individual trials.

### Data analysis

All analyses were performed in R environment (version 4.1.1) using the ‘metaumbrella’ package [45]. We re-analyzed each meta-analysis using a random-effects model with a restricted maximum likelihood estimator which was consistent with methods in previous umbrella reviews [46, 47]. Original studies reported either the mean difference (MD), raw standardized mean difference, or bias-corrected standardized mean difference (SMD). We systematically converted MD and raw standardized mean difference to SMD so that all results were reported in a similar metric to facilitate interpretation. Moreover, the direction of the effect was reversed when needed so that a positive SMD systematically reflected an improvement, i.e., a symptom reduction or a competence improvement. When CCTs included in meta-analyses reported multiple outcomes or multiple independent subgroups, we used the standard aggregating approaches [48]. When a unique group was compared to two different groups (e.g., two experimental groups compared to one control group), the resulting effect sizes were conservatively assumed to come from the same participants.

Inconsistency was assessed using I² statistics. The 95% prediction interval was computed to inform the plausible range in which the effect sizes of future studies were expected to fall. Small study effects, i.e., the tendency of the smallest studies to report significantly higher effect size estimates compared to the largest studies, were explored using the Egger’s regression asymmetry test [49, 50].

### Assessment of the credibility of the evidence

Consistent with previous umbrella reviews [51–53], we assessed the credibility of the evidence concerning the efficacy of each intervention on each outcome into five ordinal classes using an algorithmic approach: convincing (Class I), highly suggestive (Class II), suggestive (Class III), weak (Class IV), and not significant (Class ns; Table 1). Note that this analysis did not seek to generate a hierarchy leading to treatment recommendations. Instead, the aim was to summarize many statistical results into a single composite score that captured the key findings of each meta-analysis. The criteria were derived from two classification systems that usually are used for umbrella reviews and which were adapted for the specific purposes of this study (Table 1) [31, 54]. The presence of ‘small study effects’ was indicated if the *p*-value of the Egger regression test was ≤0.05. A study was considered as having a low risk of bias if the design used was a RCT and the risk of the outcome detection was low. We re-ran the assessment of the credibility of the evidence retaining only studies at low risk of bias in a sensitivity analysis to assess the robustness of the classes attributed in the primary analysis.

**Table 1:** Criteria used for the stratification of evidence.

Class	Criteria
Convincing (Class I)	▪ number of participants strictly exceeded 500 ▪ *p*-value was strictly inferior to 1e-3 ▪ inconsistency was moderate or lower (I² < 50%) ▪ 95% prediction interval excluding the null ▪ no small-study effects ▪ >75% of participants in studies at low risk of bias ▪ 5/5 critical methodological criteria of the AMSTAR-2 were met
Highly suggestive (Class II)	▪ number of participants strictly exceeded 350 ▪ *p*-value was strictly inferior to 1e-3 ▪ largest study had a statistically significant effect ▪ >50% of participants in studies at low risk of bias ▪ 4/5 critical methodological criteria of the AMSTAR-2 were met
Suggestive (Class III)	▪ number of participants strictly exceeded 200 ▪ *p*-value was strictly inferior to 1e-3 ▪ other class I–II criteria were not met
Weak (Class IV)	▪ *p*-value was strictly inferior to 5e-2 ▪ other class I–III criteria were not met
Not-significant (Class ns)	▪ *p*-value was strictly superior to 5e-2

### Data and code availability

Additional information on the results, R code supporting data analysis and raw data are publicly shared (https://corentinjgosling.github.io/MP_2022_EBIACT_PSYCHOSOCIAL).

### Creation of the EBIA-CT database

We built an open-access and interactive database that displays information on the results of the meta-analyses and information collected on the CCTs to disseminate the data that we generated during this umbrella review. The information we collected about the effect sizes, participants, interventions, and risk of bias for CCTs is available in the database. If the age range and/or the IQ range were reported but the mean age and/or the mean IQ were unknown, we imputed the mean to be equal to the median of the reported range. Certain CCTs were described in several meta-analyses due to overlapping meta-analyses. We systematically favored the information collected by the meta-analysis retained in the primary analysis in this situation. If some information was absent from the main meta-analysis but was present in another meta-analysis, this information was used as a substitute. All the results of the statistical analyses conducted for this study were included in the database. Moreover, all the information collected on the CCTs concerning the participants, intervention, and risk of bias was averaged at the meta-analysis level and displayed in the database (a weighted average by the number of participants per CCT was performed). This database will be updated at least once per year over the next five years using the same methodology as described in this manuscript.

## Results

**Fig. 1:**
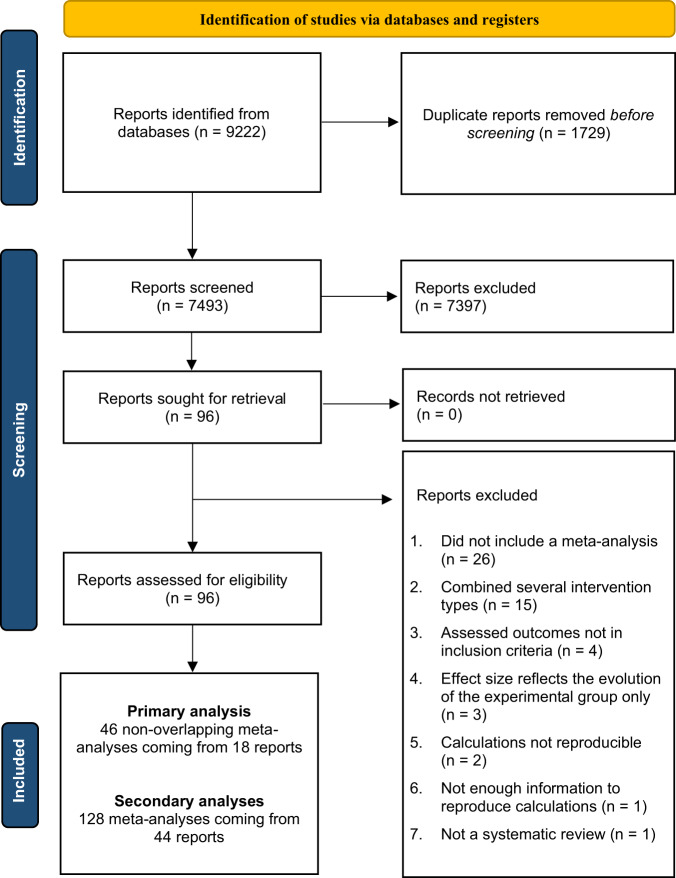
PRISMA diagram of study selection results. This figure visually summarise the screening process. More detailed information on the reasons for exclusion are available in Supplementary Materials.

A total of 7493 reports were identified initially by our systematic review (Fig. 1). Among these reports, 96 were downloaded for full-text examination and 44 were deemed eligible for this study (see Supplementary Tables S4 and S5 for the list of included and excluded studies, along with the reasons for exclusion). These 44 reports reported 128 meta-analyses (1488 effect size estimates) that were based upon more than 190 independent CCTs. The meta-analyses were categorized systematically into eight intervention subtypes: EIBI (*n* = 36), NDBI (*n* = 28), PMI (*n* = 20), TECH (*n* = 16), SSG (*n* = 15), TEACCH (*n* = 7), DEV (*n* = 4), and CBT (*n* = 2). We considered eight outcomes related to improvements in social communication deficit (*n* = 36), language deficit (*n* = 33), global cognitive abilities (*n* = 18), overall ASD symptom severity (*n* = 16), adaptive behaviors (*n* = 15), disruptive behaviors (*n* = 7), and restricted and repetitive behaviors (*n* = 3). We retained only the most recent and rigorous meta-analyses as described in the Methods section. Thus, 46 meta-analyses, derived from 18 reports, were included in the final analysis after discarding overlapping meta-analyses.

### Description of the meta-analyses included in the primary analysis

Of the 46 non-overlapping meta-analyses that were selected in the primary analysis, eight were derived from reports that met a ‘high’ methodological quality level according to the AMSTAR-2 tool while the remaining meta-analyses were derived from reports with a ‘critically low’ quality level (Supplementary Results S1.B). The main factors that lead to the downgrading of the quality ratings were the absence of either a list of excluded studies along with the reasons for exclusion, pre-registering, or a comprehensive literature search (in particular due to a restriction to articles written in English). The median number of CCTs per meta-analysis was six (interquartile range, IQR = [2, 10]), the median number of participants per meta-analysis was 238 (IQR = [68, 409]), and the median number of outcomes assessed for each intervention type was five, ranging from one to eight. The decrease of social communication symptoms was the most studied outcome. The reduction of restricted and repetitive behaviors was the least studied outcome and was targeted only in meta-analyses that explored the efficacy of NDBI and SSG.

More than half of the meta-analyses (24/46) demonstrated that participants in the experimental groups had significantly better outcomes compared to those in the control groups (i.e., a *p*-value of the pooled effect size < 0.05). Six of these 24 statistically significant meta-analyses had a pooled effect size associated with a *p*-value inferior to 1e-06, 13 were supported by a significant effect of the study with the lowest variance, and nine had a 95% prediction interval that excluded the null value. Regarding effect size magnitude, 19 meta-analyses (41%) had a moderate to large pooled effect size (SMD >= 0.50). However, a total of 12 meta-analyses we considered (*26%*) showed a moderate or large inconsistency (I² statistics superior to 50%) and we also observed small-study effects for six meta-analyses (13%). Finally, 20% of participants per meta-analysis were included in studies at ‘low risk of bias’, on average. Therefore, most of the participants included in the meta-analyses that we considered were not randomly assigned to the groups or were not assessed blindly.

### Credibility of the evidence

**Fig. 2:**
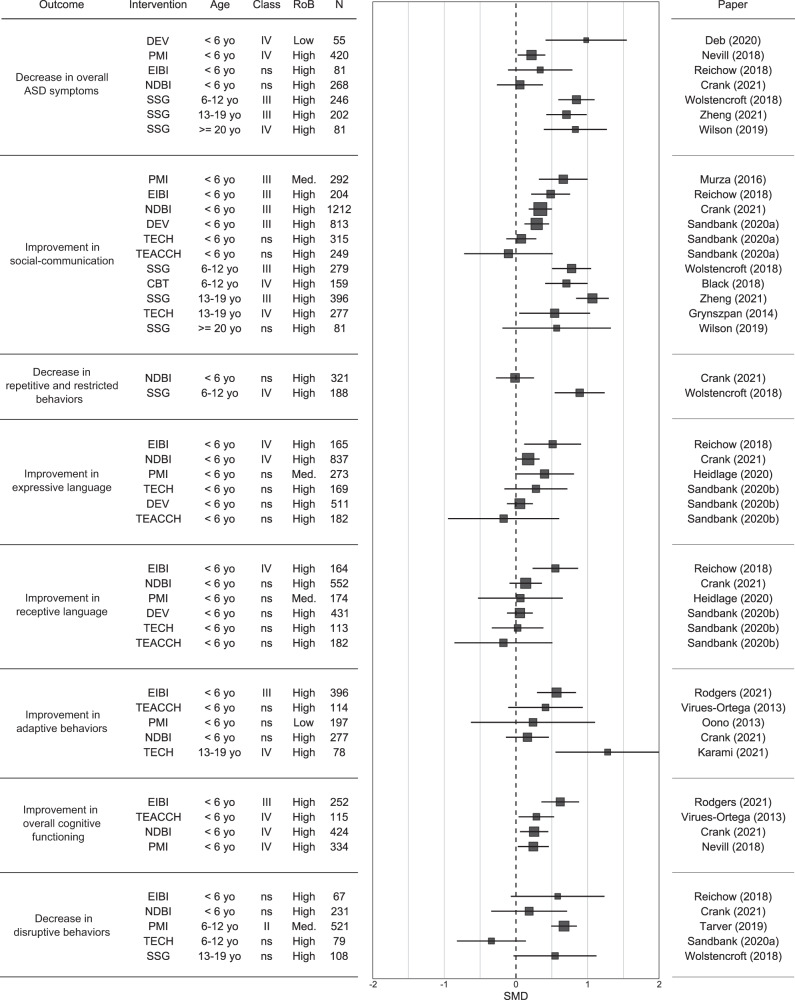
Forest plot of the pooled effect size for each intervention type and outcome we considered in the umbrella review. Some characteristics of the meta-analyses are displayed: mean age (yo = years old), class, sample size (N), and risk of bias (RoB; Low = more than 75% of the participants were in studies at low risk of bias; Med. = 50% to 75% of participants were in studies a low risk of bias; High = less than 50% of participants were in studies at low risk of bias).

Regarding core ASD symptoms, as shown in Fig. 2, EIBI, NDBI, DEV and PMI all displayed an efficacy on social communication in preschool children that was supported by suggestive evidence (Class III; Supplementary Results S1.C). The efficacy of SSG on social communication and overall ASD symptoms in school-aged children and adolescents was also supported by suggestive evidence. The efficacy of all other interventions on the overall ASD symptoms, social communication deficits or restricted/repetitive behaviors was supported by either weak or non-significant evidence (Class IV or Class ns). Regarding language skills, despite the relatively high number of meta-analyses that assessed the efficacy of psychosocial interventions on these outcomes (receptive skills: *n* = 6, and expressive skills: *n* = 6), only two interventions were supported by weak evidence: EIBI showed a significant pooled effect size on both expressive and receptive language, and NDBI on expressive language. All other interventions on language skills were supported by non-significant evidence. Regarding functional status (IQ and adaptive behaviors), these two outcomes were almost exclusively studied in preschool children, in whom EIBI showed suggestive evidence on both adaptive behaviors and IQ. All other interventions displayed either weak or non-significant evidence on these outcomes. Finally, only PMI showed a statistically significant pooled effect size on disruptive behaviors, with highly suggestive evidence in early school-aged children. Meta-analyses assessing the efficacy of EIBI, NDBI, TECH and SSG did not reach statistical significance on disruptive behaviors.

### Sensitivity analyses

A first sensitivity analysis restricted to studies at low risk of bias revealed that, only a third (*n* = 15) of the 46 meta-analyses included in our primary analysis included at least two RCTs at low risk of detection bias. Of these 15 meta-analyses, two remained with suggestive evidence (PMI on disruptive behaviors and social communication deficit in preschool children), and two remained with weak evidence (NDBI on cognition and DEV on overall ASD symptoms in preschool children). The efficacy of all other interventions was supported by non-significant evidence, even if two of these non-significant meta-analyses had a marginally significant *p*-value (Supplementary Results S2.A). Regarding the magnitude of the pooled effect sizes, whereas 41% of the meta-analyses showed a moderate-to-large pooled effect size in our primary analysis (SMD ≥ 0.50), only 20% of the meta-analyses had an effect size of this magnitude in this sensitivity analysis.

We then performed a second sensitivity analysis which gathered the meta-analyses that were excluded due to overlap. We observed that these overlapping meta-analyses reached similar conclusions in most cases, except for EIBI and NDBI in which more prominent differences occurred (Supplementary Results 2.B). For the EIBI, a first reason for the disparity in the results relates to the inclusion (or exclusion) of an RCT that compared a professional-delivered EIBI group to a parent-delivered EIBI group. In contrast to the other trials included in the meta-analyses of EIBI, this RCT provides information on the implementation modalities of EIBI rather than on the efficacy of this intervention per se [55]. Because this RCT reported very small effect sizes (if not in disfavor of the ‘intervention’ group), the meta-analyses including this trial tended to report lower pooled effect sizes compared to others. Other reasons that may explain the disparity in results include the use of different effect size measures to quantify the effects of EIBI, e.g., some meta-analyses used the differences between groups at post-test while others quantified the difference between groups for pre-post changes, different inclusion criteria for *intervention intensity*, e.g., some meta-analyses require a minimum of 10 h per week while others require more than 20 h per week, or different approaches on the *timeframe of the outcome assessment*, e.g., some meta-analyses prioritize assessments performed immediately after the intervention, while others prioritize follow-up assessments. Notably, when we replicated our primary analysis but including all the CCTs identified by all meta-analyses on EIBI, the results remained unchanged. Furthermore, divergences regarding NDBI occurred between the two main meta-analytic reports that assessed the efficacy of this intervention on overall ASD symptoms [56, 57]. The first meta-analysis found a modest but significant effect of NDBI on overall ASD symptoms (SMD = 0.38, *p*-value = 0.03) whereas the second found a very small, non-significant effect (SMD = 0.05, *p*-value = 0.75). The trials included in these two meta-analyses did not overlap fully and none of the papers provided a list of studies that were excluded from the final analysis. Interestingly, several meta-analyses of specific programs of NDBI, such as pivotal response treatment or early start Denver model, identified some trials that were not selected, or located, by the two main meta-analyses of NDBI. Thus, we repeated the calculations while including all the CCTs identified by all meta-analyses on NDBI. A total of 21 trials that assessed the efficacy of NDBI on overall ASD symptoms in preschool children were ultimately included in this reanalysis. The random-effects meta-analysis revealed a statistically significant but small pooled effect size (SMD = 0.22, *p*-value = 0.02), an efficacy that was supported by weak evidence.

## Discussion

Our umbrella review described here examined the results that were generated by a total of 128 meta-analyses. These meta-analyses synthesized the evidence provided by more than 190 unique CCTs which explored the efficacy of psychosocial interventions on core and related ASD symptoms. We observed that a substantial proportion of the meta-analyses displayed a moderate to large pooled effect size (i.e., SMD ≥ 0.50; 41% of the meta-analyses) and/or statistically significant results (53% of the meta-analyses) in favor of the psychosocial interventions. According to the algorithmic criteria developed for this umbrella review, we found that the efficacy of many of these psychosocial interventions was supported by highly suggestive (Class II) or suggestive (Class III) evidence depending on the age of the participants and the outcome under consideration. In preschool children EIBI, NDBI, DEV and PMI were supported by suggestive evidence: on social communication impairment, adaptive behaviors and IQ for EIBI, and on social communication for NDBI, PMI and DEV. In early school-aged children, highly suggestive evidence was found for the efficacy of PMI on disruptive behaviors. In late school-aged children and in adolescents, suggestive evidence was found for SSG on social communication and overall ASD symptoms. Regardless of the age of the participants, no intervention displayed an efficacy ranked as suggestive regarding either expressive or receptive language skills (EIBI and NDBI showed a significant pooled effect size but were supported by weak evidence), or repetitive and restricted behaviors (SSG had a significant pooled effect size that was supported by weak evidence). Thus, our results highlight the diversity of psychosocial approaches that are available for individuals with ASD, as well as the scientific evidence that supports the efficacy of these interventions on various outcomes at each stage of life.

A sensitivity analysis limited to RCTs with outcome assessors blinded to experimental status was conducted to complement our main results and to shed light upon the evidence generated by low risk of bias studies. This analysis revealed that a substantial proportion of the meta-analyses that showed statistically significant results in our primary analysis included less than two low risk of bias studies. Of the meta-analyses that included at least two of them, only PMI, NDBI and DEV still retained statistically significant (or marginally significant) results in preschoolers in this sensitivity analysis. The efficacy of PMI on both disruptive behaviors and social communication was ranked as suggestive (Class III), and the efficacies of both NDBI on cognition and DEV on overall ASD symptoms were ranked as weak (Class IV). No intervention was assessed by at least two low risk of bias studies in school-aged children, adolescents, or adults.

Interestingly, parallel with the decrease in statistical significance in our sensitivity analysis that was confined to RCTs with blinded outcome assessors, the proportion of effect sizes that can be considered as moderate-to-large was also smaller when only RCTs at low risk of detection bias were considered. Whereas 41% of the meta-analyses had a moderate-to-large pooled effect size in the main analysis, only 20% had a pooled effect size of this magnitude in this sensitivity analysis. This result can suggest that restricting the analyses to RCTs in which outcomes were measured by blind assessors may have discarded large pooled effect sizes derived from biased trials. However, it is important to note that by restricting to blinded outcomes, the type of outcomes included in the meta-analyses also changed. In particular, the percentage of reported outcomes decreased, while the number of outcomes assessed by standardized tests increased. Therefore, it is possible that we observed a reduction in the magnitude of the pooled effect sizes in this sensitivity analysis because psychosocial interventions produce subtle effects that can be detected in patients’ daily lives (and are therefore captured in the reports of informants seeing the patient on a day-to-day basis) but fail to be detected in more structured, less ecological assessments. Future studies may benefit from the developments of outcomes that are designed specifically to capture changes in clinical trials and longitudinal studies [58], as well as the implementation of new methodologies such as ecological momentary assessment (EMA) [59].

An additional objective of our study was to build an open-access online interactive resource (https://www.ebiact-database.com) which contains all of the information and results that we collected and generated during this umbrella review. We aimed to facilitate the dissemination of knowledge about psychosocial interventions in people with ASD by providing an open-access source of evidence-based information. This database was directly inspired by a similar project in the field of depression, i.e., the METAPSY database [60]. However, compared to this meta-analytic database, our umbrella review approach allows to provide key information not only on individual trials, but also on meta-analyses that are already published in the literature. This feature makes it possible to provide reliable information based on a meaningful pool of trials, and thus saves users from having to undertake this pooling independently (that may lead to meaningless pooling, which is a well-known problem in meta-analyses [61]).

By providing privileged access to the results of the scientific literature, we anticipate that our database will assist clinicians in keeping abreast of the most recent scientific information. We also believe that this database will be useful to researchers. This resource may be used as an interface to easily perform scoping reviews and thereby to identify gaps in knowledge about psychosocial interventions in people with ASD. It may also increase the consistency of the CCTs included in future meta-analyses because researchers will have convenient access to the list of all CCTs included in previous meta-analyses. Finally, the database will be useful to trialists. When planning new trials, few teams consider existing meta-analyses on their topic to inform the choice of materials or the power analysis [62–65]. In this regard, it has been shown that the large variability in the tools used to measure similar outcomes in the field of ASD can make direct comparisons between trials difficult [66]. The EBIA-CT database also allows the identification of the main outcome measures used in previous CCTs in a matter of minutes. Because this database contains critical information that can be used to conduct a power analysis (such as the pooled effect size estimate of a meta-analysis, its 95% prediction interval, or the effect size of the largest study), it will be useful for estimating an appropriate sample size in new trials.

A major methodological choice that we made when designing this umbrella review was assessing the credibility of evidence using algorithmic criteria rather than assessing the quality of this body of evidence using more subjective approach such as the Grading of Recommendations, Assessment, Development and Evaluations (GRADE) approach [67, 68]. In line with umbrella review guidelines [32], the main goal of the current umbrella review was to identify and appraise all of the evidence produced by meta-analyses on the efficacy of psychosocial interventions in people with ASD to guide readers to current best evidence. Our goal was not to design new recommendations for treatment or to make conclusions about the comparative efficacy of different psychosocial interventions. Instead, our context-independent algorithmic assessment of the credibility of evidence only aimed to synthesize, in a unique indicator, a large amount of information about the results of each meta-analysis, including the presence of methodological bias in primary studies, presence of small study effects, and inconsistency, using a consistent and robust method, regardless of the interventions or outcomes assessed. Such an algorithmic approach is concordant with many similar umbrella reviews of meta-analyses of clinical trials in both mental and physical health [53, 54, 69–71].

A first limitation of the present work lies in the lack of evaluation on the efficacy of specific intervention techniques (such as prompting, modelling or reinforcement) used in the different psychosocial interventions assessed in the review. In other words, we did not provide information on the efficacy of each “active ingredient” used by each psychosocial intervention type. Inclusion criteria of our systematic review allowed identification of meta-analyses that assessed the efficacy of such specific techniques, but only meta-analyses of single case experimental studies were found on this topic, e.g., the meta-analysis performed by Wang and colleagues [72]. Thus, despite the strength of the CCT design, an important direction for future research involves expanding this umbrella review to other clinical trial designs to afford a larger picture of the efficacy of psychosocial interventions in individuals with ASD. A second limitation of our study is that the information collected at the CCT-level was drawn from the meta-analyses that were included and not directly from reports describing the CCTs. This limitation, which is inherent to the umbrella review method for feasibility reasons, sometimes leads to incomplete information at the CCT-level. Future updates of the database will address the missing information by routinely providing data from new meta-analyses on the topic as they become available.

In conclusion, this umbrella review reinforced previous findings that highlighted the promising role of psychosocial interventions in individuals with ASD. However, additional well-designed RCTs are required to draw a consistent picture of the efficacy of psychosocial interventions in ASD with a higher level of evidence. The companion open-access database designed in this study will facilitate the dissemination of evidence-based knowledge about psychosocial interventions in ASD and will contribute to strengthening the methodological design of future clinical trials. This database will be regularly updated to ensure that accurate information is conveyed over time.

## Supplementary information

Supplementary Materials

## Data Availability

The datasets generated during and/or analyzed during the current study are publicly available (https://github.com/CorentinJGosling/MP_2022_EBIACT_PSYCHOSOCIAL).
