# Correction: Efficacy of psychosocial interventions for Autism spectrum disorder: an umbrella review

**DOI:** 10.1038/s41380-022-01807-0

**Published:** 2022-10-04

**Authors:** Corentin J. Gosling, Ariane Cartigny, Baptiste C. Mellier, Aleix Solanes, Joaquim Radua, Richard Delorme

**Affiliations:** 1 Paris Nanterre University, DysCo Laboratory, F-92000 Nanterre, France; 2 Université de Paris, Laboratoire de Psychopathologie et Processus de Santé, F-92100 Boulogne-Billancourt, France; 3 Centre for Innovation in Mental Health (CIMH), School of Psychology, Faculty of Environmental and Life Sciences, University of Southampton, Southampton, UK; 4 Department of Child and Adolescent Psychiatry, Robert Debré Hospital, APHP, Paris, France; 5 Imaging of Mood- and Anxiety-Related Disorders (IMARD) Group, Institut d’Investigacions Biomèdiques August Pi i Sunyer (IDIBAPS), CIBERSAM, Barcelona, Spain; 6 Department of Psychosis Studies, Institute of Psychiatry, Psychology, and Neuroscience, King’s College London, London, UK; 7 Department of Clinical Neuroscience, Centre for Psychiatric Research and Education, Karolinska Institutet, Stockholm, Sweden; 8 Human Genetics and Cognitive Functions, Institut Pasteur, Paris, France

**Keywords:** Autism spectrum disorders, Psychology

Correction to: *Molecular Psychiatry* 10.1038/s41380-022-01670-z, published online 05 July 2022

The article “Efficacy of psychosocial interventions for Autism spectrum disorder: an umbrella review”, written by Corentin J. Gosling, Ariane Cartigny, Baptiste C. Mellier, Aleix Solanes, Joaquim Radua & Richard Delorme, was originally published electronically on the publisher’s internet portal on 5 July 2022 without open access. With the authors’ decision to opt for Open Choice the copyright of the article changed on 14 September 2022 to © The Authors 2022 and the article is forthwith distributed under a Creative Commons Attribution 4.0 International License, which permits use, sharing, adaptation, distribution, and reproduction in any medium or format, as long as you give appropriate credit to the original author(s) and the source, provide a link to the Creative Commons licence, and indicate if changes were made. The images or other third-party material in this article are included in the article’s Creative Commons licence, unless indicated otherwise in a credit line to the material. If material is not included in the article’s Creative Commons licence and your intended use is not permitted by statutory regulation or exceeds the permitted use, you will need to obtain permission directly from the copyright holder. To view a copy of this licence, visit http://creativecommons.org/licenses/by/4.0/.

