# Supplementary material for: Efficacy of psychosocial interventions for Autism spectrum disorder: an umbrella review
Source: Mol Psychiatry. 2022 Jul 5;27(9):3647–56. doi: 10.1038/s41380-022-01670-z (PMC9708596; doi:10.1038/s41380-022-01670-z)
Supplement: Supplementary file 1 — Supplementary Materials [file 41380_2022_1670_MOESM1_ESM.docx]

**Supplementary Materials for "Efficacy of psychosocial interventions for Autism Spectrum Disorder: an umbrella review."**

Gosling CJ, Cartigny A, Mellier BC, Solanes A, Radua J & Delorme R

**Supplementary Table S1.** PRISMA checklist.

**Supplementary Text S2.** Deviations from protocol.

**Supplementary Text S3.** Search strategies.

**Supplementary Text S4** List of included studies.

**Supplementary Table S5.** List of excluded studies.

**Supplementary Table S1. PRISMA checklist**

Location in the manuscript of each criteria requested by the PRISMA guidelines.

| **Section and Topic** | **Item #** | **Checklist item** | **Location where item is reported** |
| --- | --- | --- | --- |
| **TITLE** | | |  |
| Title | 1 | Identify the report as a systematic review | Title page (as an umbrella review) |
| **ABSTRACT** | | |  |
| Abstract | 2 | See the PRISMA 2020 for Abstracts checklist. | p.1 |
| **INTRODUCTION** | | |  |
| Rationale | 3 | Describe the rationale for the review in the context of existing knowledge. | p.3-4 |
| Objectives | 4 | Provide an explicit statement of the objective(s) or question(s) the review addresses. | p.4 |
| **METHODS** | | |  |
| Eligibility criteria | 5 | Specify the inclusion and exclusion criteria for the review and how studies were grouped for the syntheses. | p.6 |
| Information sources | 6 | Specify all databases, registers, websites, organisations, reference lists and other sources searched or consulted to identify studies. Specify the date when each source was last searched or consulted. | p.5-6 + Supplementary materials |
| Search strategy | 7 | Present the full search strategies for all databases, registers and websites, including any filters and limits used. | Supplementary materials |
| Selection process | 8 | Specify the methods used to decide whether a study met the inclusion criteria of the review, including how many reviewers screened each record and each report retrieved, whether they worked independently, and if applicable, details of automation tools used in the process. | p.5-6 |
| Data collection process | 9 | Specify the methods used to collect data from reports, including how many reviewers collected data from each report, whether they worked independently, any processes for obtaining or confirming data from study investigators, and if applicable, details of automation tools used in the process. | p.8-9 |
| Data items | 10a | List and define all outcomes for which data were sought. Specify whether all results that were compatible with each outcome domain in each study were sought (e.g. for all measures, time points, analyses), and if not, the methods used to decide which results to collect. | p.7 |
|  | 10b | List and define all other variables for which data were sought (e.g. participant and intervention characteristics, funding sources). Describe any assumptions made about any missing or unclear information. | p.8 |
| Study risk of bias assessment | 11 | Specify the methods used to assess risk of bias in the included studies, including details of the tool(s) used, how many reviewers assessed each study and whether they worked independently, and if applicable, details of automation tools used in the process. | p.8 |
| Effect measures | 12 | Specify for each outcome the effect measure(s) (e.g. risk ratio, mean difference) used in the synthesis or presentation of results. | p.8 |
| Synthesis methods | 13a | Describe the processes used to decide which studies were eligible for each synthesis (e.g. tabulating the study intervention characteristics and comparing against the planned groups for each synthesis (item #5)). | p.9 |
|  | 13b | Describe any methods required to prepare the data for presentation or synthesis, such as handling of missing summary statistics, or data conversions. | p.9 |
|  | 13c | Describe any methods used to tabulate or visually display results of individual studies and syntheses. | p.9 |
|  | 13d | Describe any methods used to synthesize results and provide a rationale for the choice(s). If meta-analysis was performed, describe the model(s), method(s) to identify the presence and extent of statistical heterogeneity, and software package(s) used. | p.9 |
|  | 13e | Describe any methods used to explore possible causes of heterogeneity among study results (e.g. subgroup analysis, meta-regression). | p.8 |
|  | 13f | Describe any sensitivity analyses conducted to assess robustness of the synthesized results. | p.15 |
| Reporting bias assessment | 14 | Describe any methods used to assess risk of bias due to missing results in a synthesis (arising from reporting biases). | N/A |
| Certainty assessment | 15 | Describe any methods used to assess certainty (or confidence) in the body of evidence for an outcome. | Credibility of evidence, p.10 |
| **RESULTS** | | |  |
| Study selection | 16a | Describe the results of the search and selection process, from the number of records identified in the search to the number of studies included in the review, ideally using a flow diagram. | p.12 |
|  | 16b | Cite studies that might appear to meet the inclusion criteria, but which were excluded, and explain why they were excluded. | p.12 and Supplementary materials |
| Study characteristics | 17 | Cite each included study and present its characteristics. | Supplementary materials + raw data (128 individual meta analysis) |
| Risk of bias in studies | 18 | Present assessments of risk of bias for each included study. | p.13 and Supplementary materials |
| Results of individual studies | 19 | For all outcomes, present, for each study: (a) summary statistics for each group (where appropriate) and (b) an effect estimate and its precision (e.g. confidence/credible interval), ideally using structured tables or plots. | Not presented but available in raw data or in the app https://ebiact-database.com |
| Results of syntheses | 20a | For each synthesis, briefly summarise the characteristics and risk of bias among contributing studies. | p.14 |
|  | 20b | Present results of all statistical syntheses conducted. If meta-analysis was done, present for each the summary estimate and its precision (e.g. confidence/credible interval) and measures of statistical heterogeneity. If comparing groups, describe the direction of the effect. | P.14 |
|  | 20c | Present results of all investigations of possible causes of heterogeneity among study results. | N/A |
|  | 20d | Present results of all sensitivity analyses conducted to assess the robustness of the synthesized results. | p. 15-16 |
| Reporting biases | 21 | Present assessments of risk of bias due to missing results (arising from reporting biases) for each synthesis assessed. | N/A |
| Certainty of evidence | 22 | Present assessments of certainty (or confidence) in the body of evidence for each outcome assessed. | p.14 |
| **DISCUSSION** | | |  |
| Discussion | 23a | Provide a general interpretation of the results in the context of other evidence. | p.17 |
|  | 23b | Discuss any limitations of the evidence included in the review. | p.17-18 |
|  | 23c | Discuss any limitations of the review processes used. | p.19-21 |
|  | 23d | Discuss implications of the results for practice, policy, and future research. | p.19 |
| **OTHER INFORMATION** | | |  |
| Registration and protocol | 24a | Provide registration information for the review, including register name and registration number, or state that the review was not registered. | p5 |
|  | 24b | Indicate where the review protocol can be accessed, or state that a protocol was not prepared. | p5 |
|  | 24c | Describe and explain any amendments to information provided at registration or in the protocol. | Supplementary materials |
| Support | 25 | Describe sources of financial or non-financial support for the review, and the role of the funders or sponsors in the review. | p.19 |
| Competing interests | 26 | Declare any competing interests of review authors. | p.19 |
| Availability of data, code and other materials | 27 | Report which of the following are publicly available and where they can be found: template data collection forms; data extracted from included studies; data used for all analyses; analytic code; any other materials used in the review. | Everything is shared p.10 |

S**upplementary Text S2. Deviations from protocol.**

| **Registered** | **Deviation** |
| --- | --- |
| Our protocol specified that the age ranges used to present the results would be:   - preschool children (~0-6 years old), - youths (~7-21 years old) - adults (~22+ years old). | We finally decided that it was more relevant to present the results of the age more precisely. The categories used in the manuscript are:   - Preschool children : 0-5 years - School-aged children : 6-12 - Adolescents : 13-19 - Adults: 20+ |
| Our protocol specified that: *"In a sensitivity analysis, we will rerun all our analyses when considering only studies with a “non-active” control group (i.e., a waiting list, no treatment or a treatment as usual control group)"* | Unfortunately; many meta-analyses did not specify the type of control group in the different studies. Therefore, we were unable to conduct these analyses. |
| Our protocol specified that: *"Controlled clinical trials included in meta-analyses should include, at least, 5 participants per arm. A sensibility analysis, restricting to trials with at least 15 participants per group, will be run."* | Contrary to expected, very few meta-analyses included trials with fewer than 5 participants per arm. Because exclusion of trials with fewer than 15 participants per arm did not dramatically affect the results and because we already report a large number of analyses, we do not report results of this analysis for parsimony reasons. |
| Our protocol specified that: *"All outcomes will be categorized according to their proximity with the intervention target and their boundedness to the intervention context following the classification of Yoder et al. (2013). If a same construct (e.g., social-communication ability) is assessed using different methods within the same paper, the meta-analysis of the most distal and generalized outcome will be preferred."* | Contrary to expected, very few meta-analyses reported this information. Moreover, because the type of tools differs depending on the intervention studied, we did not select the meta-analyses according to the tools used. Conversely, we extracted information from all methods for the same outcome. |

**Supplementary Text S3. Search strategies.**

The search strategies used to locate systematic reviews and meta-analyses assessing the efficacy of psychosocial interventions in people with ASD. No restrictions regarding publication date or language were used

**PUBMED**
("Child Development Disorders, Pervasive"[mh] OR autis*[tw] OR asperger*[tw] OR (high functioning[tw]) OR ASD[tw] OR (Pervasive Development Disorder*[tw]) OR PDD[tw] OR HFA[tw] OR Kanner[tw]) AND (meta-analysis[pt] OR (systematic review[pt]) OR (Meta-Analysis as Topic[mh]) OR (meta analy*[tw]) OR (systematic review*[tw]) OR (Cochrane database syst rev[jour]))

**EMBASE**
('autism'/exp OR 'autis*':ti,ab,kw OR 'asperger*':ti,ab,kw OR 'high functioning':ti,ab,kw OR 'ASD':ti,ab,kw OR 'pervasive development* disorder*':ti,ab,kw OR 'PDD':ti,ab,kw OR 'HFA':ti,ab,kw OR 'Kanner':ti,ab,kw) AND (’meta-analysis’/exp OR ’systematic review’/exp OR ’meta analy*’:ti,ab,tt,kw OR ’systematic review*’:ti,ab,tt,kw OR search*:ti,ab,tt,kw)

**CENTRAL**
MeSH descriptor : [Child Development Disorders, Pervasive] explode all trees OR (autis*):ti,ab,kw OR (asperger*):ti,ab,kw OR (high functioning):ti,ab,kw OR (ASD):ti,ab,kw OR (pervasive development* disorder*):ti,ab,kw OR (PDD):ti,ab,kw OR (HFA):ti,ab,kw OR (Kanner):ti,ab,kw

**CINAHL**(MH Autistic disorder OR MH Asperger syndrome OR TI (autis*) OR AB (autis*) OR TI (asperger*) OR AB (asperger*) OR TI (high functioning) OR AB (high functioning) OR TI (ASD) OR AB (ASD) OR TI (pervasive development* disorder*) OR AB (pervasive development* disorder*) OR TI (PDD) OR AB (PDD) OR TI (HFA) OR AB (HFA) OR TI (Kanner) OR AB (Kanner)) AND (PT (systematic review) OR PT (meta analysis) OR MH (systematic review) OR MH (meta analysis) OR TI (systematic review*) OR AB (systematic review*) OR TI (meta analy*) OR AB (meta analy*))

**PSYCINFO**
(DE "Autism Spectrum Disorders" OR TI (autis*) OR AB (autis*) OR TI (asperger*) OR AB (Asperger) OR TI (high functioning) OR AB (high functioning) OR TI (ASD) OR AB(ASD) OR TI (Pervasive Development* Disorder*) OR AB (Pervasive Development* Disorder*) OR TI (PDD) OR AB (PDD) OR TI (HFA) OR AB (HFA) OR TI (Kanner) OR AB (Kanner)) AND (DE "meta analysis" OR DE "systematic review" OR TI (meta analy*) OR AB (meta analy*) OR TI (systematic review*) OR AB (systematic review*))

**Supplementary Text S4. List of included studies.**

References of all studies included in the umbrella review.

| Reference |
| --- |
| Black ME, Therrien WJ, Cook B. Parent Training Programs for School-Age Children With Autism: A Systematic Review. *Remedial & Special Education*. 2018;39(4):243-56. |
| Crank JE, Sandbank M, Dunham K, Crowley S, Bottema‐Beutel K, Feldman J, & Woynaroski TG Understanding the Effects of Naturalistic Developmental Behavioral Interventions: A Project AIM Meta‐analysis. *Autism Research* 2020 14(4), 817-834. |
| Deb SS, Retzer A, Roy M, Acharya R, Limbu B, Roy A. The effectiveness of parent training for children with autism spectrum disorder: a systematic review and meta-analyses. *BMC psychiatry* 2021*;* 20(1) : 1-24. |
| Dubreucq J, Haesebaert F, Plasse J, Dubreucq M, Franck N. A systematic review and meta-analysis of social skills training for adults with Autism Spectrum Disorder. *Journal of Autism and Developmental Disorders 2021* 1-12. |
| Eldevik S, Hastings RP, Hughes JC, Jahr E, Eikeseth S, Cross S. Meta-analysis of Early Intensive Behavioral Intervention for children with autism. *Journal of clinical child and adolescent psychology*. 2009;38(3):439-50. |
| Fuller EA, Kaiser AP. The Effects of Early Intervention on Social Communication Outcomes for Children with Autism Spectrum Disorder: A Meta-analysis. J Autism Dev Disord. 2020;50(5):1683-1700. doi:10.1007/s10803-019-03927-z |
| Fuller EA, Oliver K, Vejnoska SF, Rogers SJ. The Effects of the Early Start Denver Model for Children with Autism Spectrum Disorder: A Meta-Analysis. *Brain sciences.* 2020;10(6). |
| Gates JA, Kang E, Lerner MD. Efficacy of group social skills interventions for youth with autism spectrum disorder: A systematic review and meta-analysis. *Clinical psychology review.* 2017;52:164-81. |
| Grynszpan O, Weiss PL, Perez-Diaz F, Gal E. Innovative technology-based interventions for autism spectrum disorders: a meta-analysis. *Autism* 2014;18(4):346-61. |
| Hampton, L. H., & Kaiser, A. P. (2016). Intervention effects on spoken‐language outcomes for children with autism: a systematic review and meta‐analysis. Journal of Intellectual Disability Research, 60(5), 444-463. |
| Heidlage JK, Cunningham JE, Kaiser AP, Trivette CM, Barton EE, Frey JR, et al. The effects of parent-implemented language interventions on child linguistic outcomes: A meta-analysis. *Early Childhood Research Quarterly*. 2020; 50:6-23. |
| Karami B, Koushki R, Arabgol F, Rahmani M, & Vahabie AH. Effectiveness of Virtual/Augmented Reality-based therapeutic interventions on individuals with autism spectrum disorder: A comprehensive meta-analysis. *Frontiers in Psychiatry* 2021; 12: 887. |
| Lau HM, Smit JH, Fleming TM, Riper H. Serious games for mental health: Are they accessible, feasible, and effective? A systematic review and meta-analysis. *Frontiers in psychiatry.* 2017;7. |
| Liu Q, Hsieh W-Y, Chen G. A systematic review and meta-analysis of parent-mediated intervention for children and adolescents with autism spectrum disorder in mainland China, Hong Kong, and Taiwan. *Autism* 2020;24(8):1960-1979. |
| Makrygianni MK, Reed P. A meta-analytic review of the effectiveness of behavioural early intervention programs for children with Autistic Spectrum Disorders. *Research in autism spectrum disorders*. 2010;4(4):577-93. |
| Moon SJ, Hwang J, Hill HS, Kervin R, Birtwell KB, Torous J, et al. Mobile device applications and treatment of autism spectrum disorder: a systematic review and meta-analysis of effectiveness. Archives of disease in childhood 2020; 105(5):458-462. |
| Murza KA, Schwartz JB, Hahs‐Vaughn DL, Nye C. Joint attention interventions for children with autism spectrum disorder: a systematic review and meta-analysis. *International journal of language & communication disorders*. 2016;51(3):236-51. |
| Nevill RE, Lecavalier L, Stratis EA. Meta-analysis of parent-mediated interventions for young children with autism spectrum disorder. *Autism* 2018;22(2):84-98. |
| Ona HN, Larsen K, Nordheim LV, & Brurberg KG. Effects of Pivotal Response Treatment (PRT) for children with autism spectrum disorders (ASD): A systematic review. *Review Journal of Autism and Developmental Disorders*, 2020; *7*(1):78-90. |
| Oono IP, Honey EJ, McConachie H. Parent-mediated early intervention for young children with autism spectrum disorders (ASD). *The Cochrane database of systematic reviews*. 2013(4):Cd009774. |
| Peters-Scheffer N, Didden R, Korzilius H, Sturmey P. A meta-analytic study on the effectiveness of comprehensive ABA-based early intervention programs for children with Autism Spectrum Disorders. *Research in autism spectrum disorders*. 2011;5(1):60-9. |
| Pi HJ, Kallapiran K, Munivenkatappa S, Kandasamy P, Kirubakaran R, Russell P, & Eapen V. Meta-Analysis of RCTs of Technology-Assisted Parent-Mediated Interventions for Children with ASD. *Journal of Autism and Developmental Disorders* 2021, 1-19. |
| Postorino V, Sharp W, McCracken C, Bearss K, Burrell T, Evans A, et al. A Systematic Review and Meta-analysis of Parent Training for Disruptive Behavior in Children with Autism Spectrum Disorder. *Clinical Child & Family Psychology Review.* 2017; 20(4):391-402. |
| Ratliff-Black M, Therrien W. Parent-Mediated Interventions for School-Age Children With ASD: A Meta-Analysis. *Focus on Autism and Other Developmental Disabilities* 2020. |
| Reichow B, Steiner AM, Volkmar F. Social skills groups for people aged 6 to 21 with autism spectrum disorders (ASD). *The Cochrane database of systematic reviews*. 2012(7):Cd008511. |
| Reichow B, Hume K, Barton EE, Boyd BA. Early intensive behavioral intervention (EIBI) for young children with autism spectrum disorders (ASD). *The Cochrane database of systematic reviews*. 2018;5(5):Cd009260. |
| Rodgers M, Simmonds M, Marshall D, Hodgson R, Stewart LA, Rai D, et al. Intensive behavioural interventions based on applied behaviour analysis for young children with autism: An international collaborative individual participant data meta-analysis. *Autism 2021*; *25*(4): 1137-1153. |
| Roelofs RL, Wingbermühle E, Egger JIM, Kessels RPC. Social Cognitive Interventions in Neuropsychiatric Patients: A Meta-Analysis. *Brain Impairment.* 2017;18(1):138-73. |
| Sandbank M, Bottema-Beutel K, Crowley S, Cassidy M, Dunham K, Feldman JI, et al. Project AIM: Autism intervention meta-analysis for studies of young children. *Psychological bulletin* 2020a;146(1):1-290. |
| Sandbank M, Bottema-Beutel K, Crowley S, Cassidy M, Feldman JI, Canihuante M, et al. Intervention Effects on Language in Children With Autism: A Project AIM Meta-Analysis. *Journal of speech, language, and hearing research : JSLHR* 2020b;63(5):1537-60. |
| Shi B, Wu W, Dai M, Zeng J, Luo J, Cai L et al. Cognitive, Language, and Behavioral Outcomes in Children With Autism Spectrum Disorders Exposed to Early Comprehensive Treatment Models: A Meta-Analysis and Meta-Regression. *Frontiers in psychiatry* 2021:12. |
| Soares EE, Bausback K, Beard CL, Higinbotham M, Bunge EL, Gengoux GW. Social skills training for autism spectrum disorder: A meta-analysis of in-person and technological interventions. *Journal of Technology in Behavioral Science* 2021, *6*(1), 166-180. |
| Spreckley M, Boyd R. Efficacy of applied behavioral intervention in preschool children with autism for improving cognitive, language, and adaptive behavior: a systematic review and meta-analysis. *The Journal of pediatrics*. 2009;154(3):338-44. |
| Tang JSY, Chen NTM, Falkmer M, Blte S, Girdler S. A systematic review and meta-analysis of social emotional computer based interventions for autistic individuals using the serious game framework. *Research in autism spectrum disorders* 2019;66. |
| Tarver J, Palmer M, Webb S, Scott S, Slonims V, Simonoff E, et al. Child and parent outcomes following parent interventions for child emotional and behavioral problems in autism spectrum disorders: A systematic review and meta-analysis. *Autism* 2019;23(7):1630-44 |
| Tiede G, Walton KM. Meta-analysis of naturalistic developmental behavioral interventions for young children with autism spectrum disorder. *Autism* 2019;23(8):2080-95. |
| Virués-Ortega J. Applied behavior analytic intervention for autism in early childhood: Meta-analysis, meta-regression and dose–response meta-analysis of multiple outcomes. *Clinical psychology review*. 2010;30(4):387-99. |
| Virués-Ortega J, Julio FM, Pastor-Barriuso R. The TEACCH program for children and adults with autism: a meta-analysis of intervention studies. *Clinical psychology review.* 2013;33(8):940-53. |
| Wang Z, Loh SC, Tian J, & Chen QJ. A meta-analysis of the effect of the Early Start Denver Model in children with autism spectrum disorder. International Journal of Developmental Disabilities 2021 1-11. |
| Wilson KP, Steinbrenner JR, Kalandadze T, Handler L. Interventions Targeting Expressive Communication in Adults With Autism Spectrum Disorders: A Systematic Review. *Journal of speech, language, and hearing research : JSLHR*. 2019;62(6):1959-78. |
| Wolstencroft J, Robinson L, Srinivasan R, Kerry E, Mandy W, Skuse D. A Systematic Review of Group Social Skills Interventions, and Meta-analysis of Outcomes, for Children with High Functioning ASD. *Journal of autism and developmental disorders.* 2018; 48(7):2293-307. |
| Zhang Q, Wu R, Zhu S, Le J, Chen Y, Lan C, et al. Facial emotion training as an intervention in autism spectrum disorder: A meta‐analysis of randomized controlled trials. *Autism Research* 2021, 14(10), 2169-2182. |
| Zheng S, Kim H, Salzman E, Ankenman K, & Bent S. Improving social knowledge and skills among adolescents with autism: systematic review and meta-analysis of UCLA PEERS® for adolescents. *Journal of Autism and Developmental Disorders* 2021, 1-16. |
| Zhi W., Cheong L.S., & Jing T. (2021). Meta-Analysis of Emotion Recognition Intervention Effects and Influencing Factors in Autism Spectrum Disorder. *Education and Training in Autism and Developmental Disabilities*, *56*(4), 479-493. |

**Supplementary Table S5. List of excluded studies.**

References of all studies excluded from the umbrella review along with the reason for exclusion.

| Reference | Reason for exclusion |
| --- | --- |
| Park, E. Y., Kim, J., & Kim, S. S. (2016). Meta-analysis of the effect of job-related social skill training for secondary students with disabilities. Journal of Vocational Rehabilitation, 44(1), 123-133. | Assessed outcomes not in inclusion criteria |
| Lamb, R., Miller, D., Lamb, R., Akmal, T., & Hsiao, Y. J. (2018). Examination of the role of training and fidelity of implementation in the use of assistive communications for children with autism spectrum disorder: a meta‐analysis of the Picture Exchange Communication System. British Journal of Special Education, 45(4), 454-472. | Assessed outcomes not in inclusion criteria |
| Khan, K., Hall, C. L., Davies, E. B., Hollis, C., & Glazebrook, C. (2019). The effectiveness of web-based interventions delivered to children and young people with neurodevelopmental disorders: systematic review and meta-analysis. Journal of medical Internet research, 21(11), e13478. | Assessed outcomes not in inclusion criteria |
| Perihan, C., Burke, M., Bowman-Perrott, L., Bicer, A., Gallup, J., Thompson, J., & Sallese, M. (2020). Effects of cognitive behavioral therapy for reducing anxiety in children with high functioning ASD: a systematic review and meta-analysis. Journal of autism and developmental disorders, 50(6), 1958-1972. | Assessed outcomes not in inclusion criteria |
| Sandgreen, H., Frederiksen, L. H., & Bilenberg, N. (2020). Digital interventions for autism spectrum disorder: a meta-analysis. Journal of Autism and Developmental Disorders, 1-15. | Calculations not reproducible |
| Yu, Q., Li, E., Li, L., & Liang, W. (2020). Efficacy of interventions based on applied behavior analysis for autism spectrum disorder: A meta-analysis. Psychiatry investigation, 17(5), 432. | Calculations not reproducible |
| Dunst, C. J., Trivette, C. M., & Hamby, D. W. (2012). Meta-analysis of studies incorporating the interests of young children with autism spectrum disorders into early intervention practices. Autism research and treatment:462531 | Combined several intervention types |
| Fletcher‐Watson, S., McConnell, F., Manola, E., & McConachie, H. (2014). Interventions based on the Theory of Mind cognitive model for autism spectrum disorder (ASD). Cochrane Database of Systematic Reviews, (3). | Combined several intervention types |
| Ahn, S. N., & Hwang, S. (2018). Cognitive rehabilitation of adaptive behavior in children with neurodevelopmental disorders: a meta-analysis. Occupational Therapy International, 2018. | Combined several intervention types |
| Naveed, S., Waqas, A., Amray, A. N., Memon, R. I., Javed, N., Tahir, M. A., ... & Rahman, A. (2019). Implementation and effectiveness of non-specialist mediated interventions for children with Autism Spectrum Disorder: A systematic review and meta-analysis. PloS one, 14(11), e0224362. | Combined several intervention types |
| Kent, C., Cordier, R., Joosten, A., Wilkes-Gillan, S., Bundy, A., & Speyer, R. (2020). A systematic review and meta-analysis of interventions to improve play skills in children with Autism Spectrum Disorder. Review Journal of Autism and Developmental Disorders, 7(1), 91-118. | Combined several intervention types |
| McDaniel, J., Brady, N. C., & Warren, S. F. (2021). Effectiveness of Responsivity Intervention Strategies on Prelinguistic and Language Outcomes for Children with Autism Spectrum Disorder: A Systematic Review and Meta-Analysis of Group and Single Case Studies. Journal of Autism and Developmental Disorders, 1-34. | Combined several intervention types |
| Zhang, Q., Wu, R., Zhu, S., Le, J., Chen, Y., Lan, C., ... & Kendrick, K. M. (2021). Facial emotion training as an intervention in autism spectrum disorder: A meta‐analysis of randomized controlled trials. Autism Research, 14(10), 2169-2182. | Combined several intervention types |
| Ospina, M. B., Krebs Seida, J., Clark, B., Karkhaneh, M., Hartling, L., Tjosvold, L., ... & Smith, V. (2008). Behavioural and developmental interventions for autism spectrum disorder: a clinical systematic review. PloS one, 3(11), e3755. | Combined several intervention types |
| Weston, L., Hodgekins, J., & Langdon, P. E. (2016). Effectiveness of cognitive behavioural therapy with people who have autistic spectrum disorders: A systematic review and meta-analysis. Clinical psychology review, 49, 41-54. | Combined several intervention types |
| Parsons, L., Cordier, R., Munro, N., Joosten, A., & Speyer, R. (2017). A systematic review of pragmatic language interventions for children with autism spectrum disorder. PloS one, 12(4), e0172242. | Combined several intervention types |
| Tachibana, Y., Miyazaki, C., Ota, E., Mori, R., Hwang, Y., Kobayashi, E., ... & Kamio, Y. (2017). A systematic review and meta-analysis of comprehensive interventions for pre-school children with autism spectrum disorder (ASD). PloS one, 12(12), e0186502. | Combined several intervention types |
| Maw, S. S., & Haga, C. (2018). Effectiveness of cognitive, developmental, and behavioural interventions for Autism Spectrum Disorder in preschool-aged children: A systematic review and meta-analysis. Heliyon, 4(9), e00763. | Combined several intervention types |
| Tachibana, Y., Miyazaki, C., Mikami, M., Ota, E., Mori, R., Hwang, Y., ... & Kamio, Y. (2018). Meta-analyses of individual versus group interventions for pre-school children with autism spectrum disorder (ASD). PloS one, 13(5), e0196272. | Combined several intervention types |
| Bejarano-Martín, Á., Canal-Bedia, R., Magán-Maganto, M., Fernández-Álvarez, C., Lóa-Jónsdóttir, S., Saemundsen, E., ... & Posada, M. (2020). Efficacy of focused social and communication intervention practices for young children with autism spectrum disorder: A meta-analysis. Early Childhood Research Quarterly, 51, 430-445. | Combined several intervention types |
| Kent, C., Cordier, R., Joosten, A., Wilkes-Gillan, S., Bundy, A., & Speyer, R. (2020). A systematic review and meta-analysis of interventions to improve play skills in children with Autism Spectrum Disorder. Review Journal of Autism and Developmental Disorders, 7(1), 91-118. | Combined several intervention types |
| Wang, P., & Spillane, A. (2009). Evidence-based social skills interventions for children with autism: A meta-analysis. Education and Training in Developmental Disabilities, 318-342. | Did not include a meta-analysis (any or of group design) |
| Flippin, M., Reszka, S., & Watson, L. R. (2010). Effectiveness of the Picture Exchange Communication System (PECS) on communication and speech for children with autism spectrum disorders: A meta-analysis. | Did not include a meta-analysis (any or of group design) |
| Sagayaraj, K., Gopal, C. R., & Karthikeyan, S. (2020). The efficacy of technology and non-technology based intervention for children with autism spectrum disorder: a meta-analysis. International journal of Innovative Science and Research Technology, 5(3), 863-868. | Did not include a meta-analysis (any or of group design) |
| Bene, K., & Lapina, A. (2021). A meta-analysis of sibling-mediated intervention for brothers and sisters who have autism spectrum disorder. Review Journal of Autism and Developmental Disorders, 8(2), 186-194. | Did not include a meta-analysis (any or of group design) |
| Speyer, R., Chen, Y. W., Kim, J. H., Wilkes-Gillan, S., Nordahl-Hansen, A. J., Wu, H. C., & Cordier, R. (2021). Non-pharmacological interventions for adults with autism: a systematic review of randomised controlled trials. Review Journal of Autism and Developmental Disorders, 1-31. | Did not include a meta-analysis (any or of group design) |
| Schlosser, R. W., & Wendt, O. (2008). Effects of augmentative and alternative communication intervention on speech production in children with autism: A systematic review. | Did not include a meta-analysis (any or of group design) |
| Howlin, P., Magiati, I., & Charman, T. (2009). Systematic review of early intensive behavioral interventions for children with autism. American journal on intellectual and developmental disabilities, 114(1), 23-41. | Did not include a meta-analysis (any or of group design) |
| Tanner, K., Hand, B. N., O’Toole, G., & Lane, A. E. (2015). Effectiveness of interventions to improve social participation, play, leisure, and restricted and repetitive behaviors in people with autism spectrum disorder: A systematic review. American Journal of Occupational Therapy, 69(5), 6905180010p1-6905180010p12. | Did not include a meta-analysis (any or of group design) |
| Bond, C., Symes, W., Hebron, J., Humphrey, N., Morewood, G., & Woods, K. (2016). Educational interventions for children with ASD: A systematic literature review 2008–2013. School Psychology International, 37(3), 303-320. | Did not include a meta-analysis (any or of group design) |
| Chang, Y. C., & Locke, J. (2016). A systematic review of peer-mediated interventions for children with autism spectrum disorder. Research in autism spectrum disorders, 27, 1-10. | Did not include a meta-analysis (any or of group design) |
| Baril, E. M., & Humphreys, B. P. (2017). An evaluation of the research evidence on the Early Start Denver Model. Journal of Early Intervention, 39(4), 321-338. | Did not include a meta-analysis (any or of group design) |
| Spain, D., Sin, J., Paliokosta, E., Furuta, M., Prunty, J. E., Chalder, T., ... & Happe, F. G. (2017). Family therapy for autism spectrum disorders. Cochrane Database of Systematic Reviews, (5). | Did not include a meta-analysis (any or of group design) |
| Ameis, S. H., Kassee, C., Corbett‐Dick, P., Cole, L., Dadhwal, S., Lai, M. C., ... & Correll, C. U. (2018). Systematic review and guide to management of core and psychiatric symptoms in youth with autism. Acta Psychiatrica Scandinavica, 138(5), 379-400. | Did not include a meta-analysis (any or of group design) |
| Watkins, L., Ledbetter-Cho, K., O'Reilly, M., Barnard-Brak, L., & Garcia-Grau, P. (2019). Interventions for students with autism in inclusive settings: A best-evidence synthesis and meta-analysis. Psychological Bulletin, 145(5), 490. | Did not include a meta-analysis (any or of group design) |
| Carruthers, S., Pickles, A., Slonims, V., Howlin, P., & Charman, T. (2020). Beyond intervention into daily life: A systematic review of generalisation following social communication interventions for young children with autism. Autism Research, 13(4), 506-522. | Did not include a meta-analysis (any or of group design) |
| Chancel, R., Miot, S., Dellapiazza, F., & Baghdadli, A. (2020). Group-based educational interventions in adolescents and young adults with ASD without ID: a systematic review focusing on the transition to adulthood. European Child & Adolescent Psychiatry, 1-21. | Did not include a meta-analysis (any or of group design) |
| Dandil, Y., Smith, K., Kinnaird, E., Toloza, C., & Tchanturia, K. (2020). Cognitive remediation interventions in autism spectrum condition: A systematic review. Frontiers in Psychiatry, 11, 722. | Did not include a meta-analysis (any or of group design) |
| Dawson-Squibb, J. J., Davids, E. L., Harrison, A. J., Molony, M. A., & de Vries, P. J. (2020). Parent Education and Training for autism spectrum disorders: Scoping the evidence. Autism, 24(1), 7-25. | Did not include a meta-analysis (any or of group design) |
| Koegel, L. K., Bryan, K. M., Su, P. L., Vaidya, M., & Camarata, S. (2020). Parent education in studies with nonverbal and minimally verbal participants with autism spectrum disorder: A systematic review. American journal of speech-language pathology, 29(2), 890-902. | Did not include a meta-analysis (any or of group design) |
| Lake, J. K., Tablon Modica, P., Chan, V., & Weiss, J. A. (2020). Considering efficacy and effectiveness trials of cognitive behavioral therapy among youth with autism: A systematic review. Autism, 24(7), 1590-1606. | Did not include a meta-analysis (any or of group design) |
| McKeithan, G. K., & Sabornie, E. J. (2020). Social–behavioral interventions for secondary-level students with high-functioning autism in public school settings: A meta-analysis. Focus on Autism and Other Developmental Disabilities, 35(3), 165-175. | Did not include a meta-analysis (any or of group design) |
| Muharib, R., & Lang, R. (2020). Systematic review suggests social-communication interventions can be effective when implemented in inclusive schools with children with autism spectrum disorders1. Evidence-Based Communication Assessment and Intervention, 14(3), 109-112. | Did not include a meta-analysis (any or of group design) |
| Shalev, R. A., Lavine, C., & Di Martino, A. (2020). A systematic review of the role of parent characteristics in parent-mediated interventions for children with autism spectrum disorder. Journal of Developmental and Physical Disabilities, 32(1), 1-21. | Did not include a meta-analysis (any or of group design) |
| Tupou, J. (2020). Meta-analysis supports naturalistic developmental behavioral interventions as a promising approach for improving a range of outcomes for children with autism spectrum disorder. Evidence-Based Communication Assessment and Intervention, 14(4), 206-210. | Did not include a meta-analysis (any or of group design) |
| Valentine, A. Z., Brown, B. J., Groom, M. J., Young, E., Hollis, C., & Hall, C. L. (2020). A systematic review evaluating the implementation of technologies to assess, monitor and treat neurodevelopmental disorders: A map of the current evidence. Clinical psychology review, 80, 101870. | Did not include a meta-analysis (any or of group design) |
| Wang, D., Mason, R. A., Lory, C., Kim, S. Y., David, M., & Guo, X. (2020). Vocal Stereotypy and Autism Spectrum Disorder: A Systematic Review of Interventions. Research in Autism Spectrum Disorders, 78, 101647. | Did not include a meta-analysis (any or of group design) |
| Reichow, B., & Wolery, M. (2009). Comprehensive synthesis of early intensive behavioral interventions for young children with autism based on the UCLA young autism project model. Journal of autism and developmental disorders, 39(1), 23-41. | Effect size reflects the evolution of the experimental group only |
| Makrygianni, M. K., Gena, A., Katoudi, S., & Galanis, P. (2018). The effectiveness of applied behavior analytic interventions for children with Autism Spectrum Disorder: A meta-analytic study. Research in Autism Spectrum Disorders, 51, 18-31. | Effect size reflects the evolution of the experimental group only |
| Nahmias, A. S., Pellecchia, M., Stahmer, A. C., & Mandell, D. S. (2019). Effectiveness of community‐based early intervention for children with autism spectrum disorder: a meta‐analysis. Journal of Child Psychology and Psychiatry, 60(11), 1200-1209. | Effect size reflects the evolution of the experimental group only |
| Strauss, K., Mancini, F., Fava, L., & SPC Group. (2013). Parent inclusion in early intensive behavior interventions for young children with ASD: A synthesis of meta-analyses from 2009 to 2011. Research in developmental disabilities, 34(9), 2967-2985. | Not a systematic review |
| Barton, E. E., Pustejovsky, J. E., Maggin, D. M., & Reichow, B. (2017). Technology-aided instruction and intervention for students with ASD: A meta-analysis using novel methods of estimating effect sizes for single-case research. Remedial and Special Education, 38(6), 371-386. | Not enough information to reproduce calculations |
